# miR-181c-5p mediates apoptosis of vascular endothelial cells induced by hyperoxemia via ceRNA crosstalk

**DOI:** 10.1038/s41598-021-95712-1

**Published:** 2021-08-16

**Authors:** Jizhi Wu, Guangqi Zhang, Hui Xiong, Yuguang Zhang, Gang Ding, Junfeng Ge

**Affiliations:** 1 Department of Anesthesiology, Shandong Second Provincial General Hospital, Jinan, Shandong People’s Republic of China; 2 Department of Anesthesiology, Jinan Second People’s Hospital, No. 148 Jingyi Road, Jinan, 250021 Shandong People’s Republic of China; 3 Department of Pediatric Oncology, Shandong Cancer Hospital and Institute, Shandong First Medical University and Shandong Academy of Medical Sciences, Jinan, Shandong People’s Republic of China; 4 Eye Reseach Institute, Jinan Eye Hospital, Jinan, Shandong People’s Republic of China; 5 Ophthalmology, Jinan Eye Hospital, Jinan, Shandong People’s Republic of China

**Keywords:** Cardiology, Diseases, Pathogenesis, Signs and symptoms

## Abstract

Oxygen therapy has been widely used in clinical practice, especially in anesthesia and emergency medicine. However, the risks of hyperoxemia caused by excessive O_2_ supply have not been sufficiently appreciated. Because nasal inhalation is mostly used for oxygen therapy, the pulmonary capillaries are often the first to be damaged by hyperoxia, causing many serious consequences. Nevertheless, the molecular mechanism by which hyperoxia injures pulmonary capillary endothelial cells (LMECs) has not been fully elucidated. Therefore, we systematically investigated these issues using next-generation sequencing and functional research techniques by focusing on non-coding RNAs. Our results showed that hyperoxia significantly induced apoptosis and profoundly affected the transcriptome profiles of LMECs. Hyperoxia significantly up-regulated miR-181c-5p expression, while down-regulated the expressions of NCAPG and lncRNA-DLEU2 in LMECs. Moreover, LncRNA-DLEU2 could bind complementarily to miR-181c-5p and acted as a miRNA sponge to block the inhibitory effect of miR-181c-5p on its target gene NCAPG. The down-regulation of lncRNA-DLEU2 induced by hyperoxia abrogated its inhibition of miR-181c-5p function, which together with the hyperoxia-induced upregulation of miR-181c-5p, all these significantly decreased the expression of NCAPG, resulting in apoptosis of LMECs. Our results demonstrated a ceRNA network consisting of lncRNA-DLEU2, miR-181c-5p and NCAPG, which played an important role in hyperoxia-induced apoptosis of vascular endothelial injury. Our findings will contribute to the full understanding of the harmful effects of hyperoxia and to find ways for effectively mitigating its deleterious effects.

## Introduction

Oxygen therapy using high-concentration or pure oxygen is a widely used therapeutic technique to correct hypoxemia in anesthesia and emergency medicine^1^. However, because of its strong oxidizing properties, the excessive O_2_ supply, namely hyperoxia, will lead to over high arterial O_2_ tension (PaO_2_) and even hyperoxemia and oxygen poisoning. Hyperoxemia can impair various bodily functions including the lungs, brain, kidneys, and heart, resulting in hypotensive shock, and even disseminated intravascular coagulation (DIC), which can lead to death^2^. Many clinical studies have suggested that hyperoxemia is associated with worse outcomes and increased mortality in some critically ill patients^3–7^. Nonetheless, the risks of hyperoxemia have always been overlooked by clinicians and anesthesiologists compared with hypoxemia. Moreover, the mechanisms of respiratory and systemic injury caused by hyperoxemia also remain poorly understood.

The increased reactive oxygen species (ROS) levels caused by excess O_2_ administration is the generally accepted mechanism of oxygen toxicity in hyperoxemia^3,8^. The capillary endothelial cell injury induced by ROS leads to microcirculation disorders, hypotension, shock or even DIC^9^. These pathologies are important contributors to the damage of heart, brain, lung, and kidney, and even death caused by hypoxemia. The oxidative stress induced by excessive ROS is one of the key factors of vascular endothelial cells injury and death^10–12^. Therefore, the in-depth study of the mechanisms by which ROS damage the vascular endothelium is important for elucidating the mechanism of oxygen toxicity in hyperoxemia and finding effective means of prevention and treatment.

MiRNAs are a class of small non-coding RNAs and involved in the regulation of almost all endothelial functions^13^. Moreover, there are complex inter-regulations between ROS and miRNAs. On the one hand, miRNA can regulate cellular redox homeostasis and ROS production^14^. On the other hand, ROS can also regulate the expression of miRNAs and their functions through multiple mechanisms. Some pro-apoptotic miRNAs, such as miR-200 family, can be up-regulated by ROS and suppress endothelial cell proliferation by inhibiting the expression of target mRNAs, thereby promoting apoptosis and endothelial cell senescence^15^. However, some other ROS-induced miRNAs, such as miR-210, have anti-apoptotic functions and protect human umbilical vein endothelial cells (HUVEC) from apoptosis induced by oxidative stress^16^. Obviously, the regulatory relationship among ROS, miRNA and apoptosis are very complicated. Especially, the roles and mechanisms of miRNAs in the endothelial injury caused by hyperoxic oxidative stress, which is closely related to anesthesia, are still not fully elucidated.

LncRNAs are long non-coding RNAs that play essential roles in the functions of endothelial cells including apoptosis^17,18^. Many lncRNAs have miRNA recognition elements (MRE) specific to certain miRNAs and suppress the function of these mRNAs just like miRNA sponges. This crosstalk between lncRNAs and miRNAs is called the ‘competitive endogenous RNA (ceRNA)’ network and has been supported by numerous studies^17,18^. The expressions of lncRNAs in hyperoxia-induced bronchopulmonary dysplasia (BPD) were found to be changed significantly^19^, which indicated that lncRNA was closely associated with the pathologies caused by hyperoxia. However, the studies on the role and mechanism of the lncRNA and ceRNA network in the hyperoxia-induced vascular endothelial cell injury are still in their infancy.

Herein, we systematically investigated the effects of hyperoxia on the transcriptome of lung capillary endothelial cells (LMECs) including mRNA, miRNA and lncRNA using next generation sequencing. Our findings showed that hyperoxia profoundly affected the transcriptome characteristics of LMECs and changed many important biological functions and signaling pathways. Especially, our results revealed for the first time the role of a ceRNA consisting of lncRNA-DLEU2, miR-181c-5p and NCAPG in vascular endothelial damage caused by hyperoxia.

## Material and methods

### Cell culture, vectors and treatment

Human lung microvascular endothelium cells (CRL-3244, LMECs) were purchased from American Type Culture Collection (ATCC) and were cultured in conditions recommended by ATCC. The siRNA, shRNA and expression vector of NCAPG and lnc- DLEU2 were obtained from Origene or GenePharma (Shanghai, China). The mimic and inhibitor of miR-181c-5p as well as corresponding negative control sequences were bought from GenePharma (Shanghai, China). When the confluence of cells reached about 70–80%, LMECs were treated at normoxic (21% O_2_/5% CO_2_/74% N_2_) or hyperoxic (80% O_2_/5% CO_2_/15% N_2_) conditions for 0 h, 12 h and 24 h. The mimic and inhibitor of miR-181c-5p or different vectors, respectively, were transfected into LMECs using lipofectamine RNAIMAX or 3000 reagent (Life Technologies, Carlsbad, CA, USA) according to the manufacturer's instructions. These HUVECs with different treatments were harvested for subsequent experiments.

### RNA isolation, RNA sequencing and data analysis

Total RNA was isolated from LMECs with different treatments using Trizol reagent and were quantified by Nanodrop 2000 spectrophotometer (Thermo Scientific, Carlsbad, CA, USA). Then, Deep sequencing was performed on Illumina Hiseq X Ten (Illumina, San Diego, USA; SE50 model). The expression matrix of mRNA, lncRNA and miRNA was obtained using R package bowtie2 (version 2.0.6) and miRBase v21. The differentially expressed mRNAs, lncRNAs and miRNAs were identified using DESeq2 (Version 1.4.5). DAVID 6.8 and Enrichr were used to perform functional enrichment analysis. Target transcripts (mRNAs and lncRNAs) of miR-181c-5p were predicated by Targetscan, MicroT-CDS and miRANda. The folding energy (FE) for heteroduplex was calculated by RNA22 v2 and the positive threshold of binding sites was set to FE < − 12 kcal/mol^20^. Based on the predicted targets (mRNAs and lncRNAs) of miR-181c-5p, the correlation networks of lncRNAs and mRNAs (Pearson correlation coefficient > 0.90, corrected *P* < 0.01) potentially targeted by miR-181c-5p were constructed by STRING database 11.0 and a cytoscape plug (ExpressionCorrelation)^21^. The topological structure and core subnetworks were analyzed using Cytoscape software.

### Quantitative real-time PCR (qRT-PCR)

PrimeScript RT Master Mix and TB Green Premix Ex Taq II (Takara, Kusatsu, Japan) were used for cDNA synthesis and PCR amplification. GAPDH was used as the internal standard. The sequences of all primers for gene expression analysis were listed in Table 1. Mir-X miRNA First-Strand Synthesis Kit and Mir-X miRNA qRT-PCR TB Green Kit (Clotech, Kusatsu, Japan) were used for miRNA-specific cDNA synthesis and PCR amplification. MiRNA expression was normalized to snRNA u6. The sequences of all miRNA primers were purchased from Takara. PCR was performed using Bio-Rad iQ5 system. The 2^−ΔΔCt^ method was used to calculate the relative expression levels of mRNAs, lncRNAs and miRNAs.

**Table 1:** qRT-PCR primers used in this study.

Genes	Primer sequences
**lncRNA-DLEU2**	
Forward	5′-TCTGGAGAACAGCCTCACTTC-3′
Reverse	5′-TGCTGAGCTAAGTAGAGGTCTC-3′
**NCAPG**	
Forward	5′-GACGAACAGGAGGTGTCAGACT-3′
Reverse	5′-TGCTGCGGTTTTGGCTCGTCTT-3′
**GAPDH**	
Forward	5′-GGAGCGAGATCCCTCCAAAAT-3′
Reverse	5′-GGCTGTTGTCATACTTCTCATGG-3′

### Apoptosis assay using Annexin V/propidium iodide staining

LMECs with different treatment were stained by Annexin V/propidium iodide staining (BD Pharmingen, San Jose, USA), and then were analyzed using BD FACSCalibur following the manufacturer’s instructions.

### Luciferase reporter assay

The luciferase reporter vectors were constructed by cloning the sequences of the wild or mutated miR-181c-5p binding sites in NCAPG 3′UTR or in lnc-DLEU2 into the pmirGLO vector (E1330, Promega, Madison, WI, USA). LMECs, respectively, were transfected with these vectors and miR-181c-5p mimic/inhibitor or corresponding negative control sequences. The firefly and renilla luciferase activity of cell lysates were assayed by Dual-Luciferase Reporter Assay System using a Centro XS^3^ LB 960 Microplate Luminometer (Berthold, Bad Wildbad, Germany) according to the manufacturer’s manual.

### Western blotting

LMECs with different treatments were lysed in the cold lysis buffer (50 mM Tris (pH 7.5), 2 mM EDTA, 100 mM NaCl, 50 mM NaF, 1% Triton X-100, 1 mM Na_3_VO_4_, and 40 mM β-glycerol phosphate) containing a protease inhibitor cocktail (Roche, Mannheim, Germany). The concentration of purified protein was determined by BCA assay (Beyotime, Shanghai, China). The extracts were separated by SDS-PAGE and electro-transferred to polyvinylidene difluoride membrane (BioRad, Berkeley, USA). The membrane was blocked with 5% nonfat milk and then incubated with anti-NCAPG or anti-β-actin primary antibody and anti-rabbit secondary antibodies (Abcam, Cambridge, MA, USA). Protein bands were visualized using a ChemiDoc XRS + imaging system (Bio-Rad, Hercules, USA). Densitometry analysis was performed using Image pro plus 6.0 software (Media Cybernetics, Rockville, USA).

### Statistical analysis

Data normality was assessed with the Shapiro–Wilk test. Data conforming to a normal distribution were tested with two-tailed Student's t-test and one-way ANOVA test along with appropriate post hoc tests for two and multiple group comparisons, respectively, for non-normally distributed data, Kruskal–Wallis test with Dunnett's post hoc test was used for multiple group comparisons. Functional enrichment analysis was performed using Fisher's exact test with Benjamini–Hochberg FDR multiple test correction. For statistical analysis of the sequencing data, the Kruskal–Wallis test adjusted by the Benjamini–Hochberg method with FDR < 5% referring to previous reports^22^. GraphPad Prism 8.1 (GraphPad Software, San Diego, USA) was used for the bar charts and corresponding statistical analysis. The bubble charts and heat maps were drawn with R package (R-4.0.0 Version, https://www.R-project.org)^23^. The *p*  <  0.05 or adjusted *p* value < 0.05 was deemed statistically significant. All experiments were repeated independently at least three times.

## Results

### Expression profiles and functional enrichment analysis of mRNAs, lncRNAs and miRNAs in human LMECs treated by high-concentration oxygen

**Figure 1:**
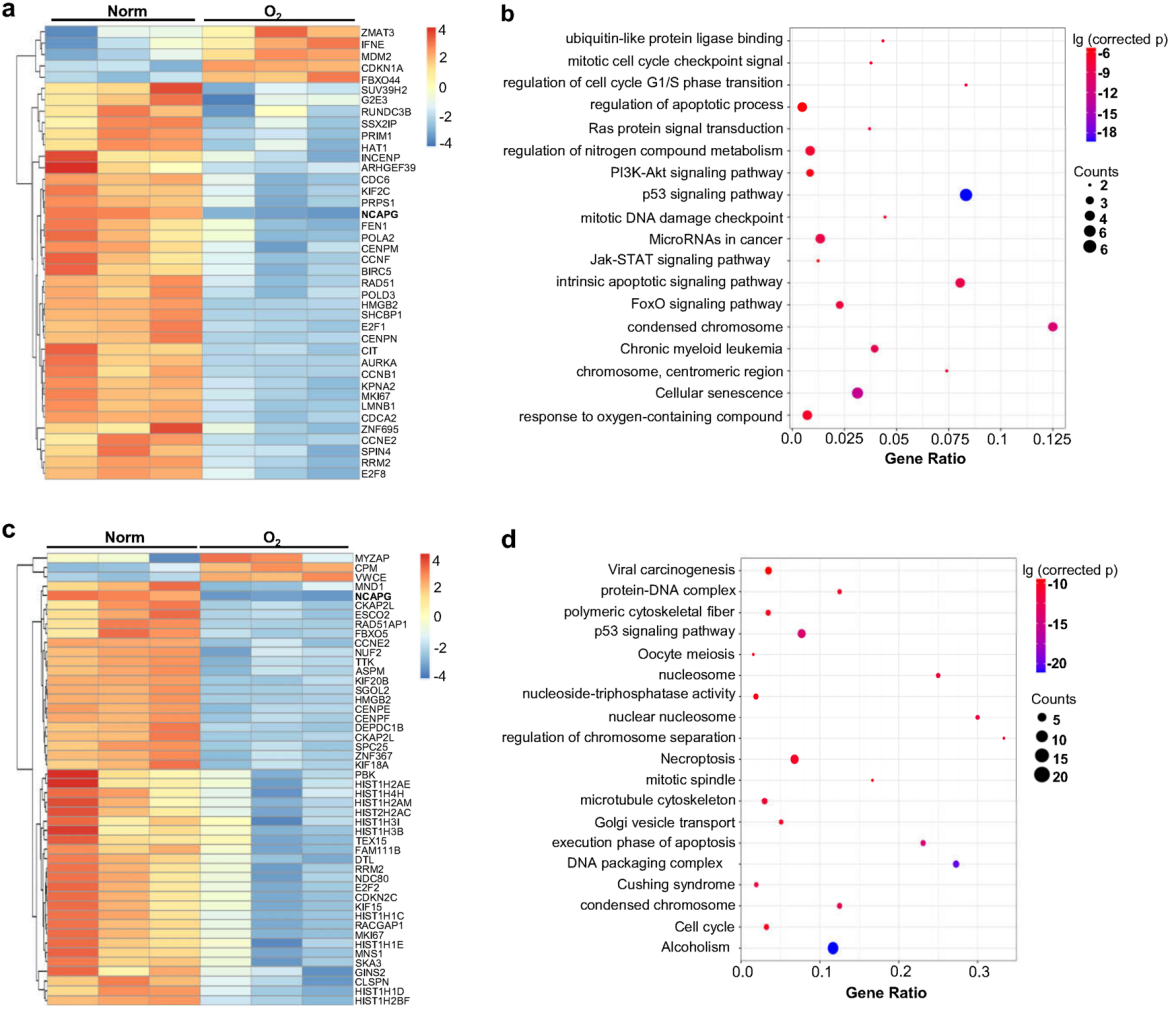
Significantly changed profiles of expression and function induced by hyperoxia in human LMECs. (**a**) Heatmap of the standardized expression levels of the potential target genes of the significantly different miRNAs. These potential target genes were predicated as described in “Material and Methods”. Then, the intersection between these predicated target genes and the significantly differentially expressed mRNAs were selected. Next, the mRNAs in this intersection that were significantly negatively correlated with the levels of miRNA expression (corrected *p* < 0.05, R < − 0.5) were considered as the potential target genes of the significantly differentially expressed miRNAs. (**b**) Enrichment analysis of the potential target genes of the significantly differentially expressed miRNAs. (**c**) Heatmap of the standardized expression levels of mRNAs that may be regulated by significantly different lncRNAs. The significantly differentially expressed mRNAs that were significantly correlated with the expression levels of lncRNAs (corrected *p* < 0.05, |R|> 0.5) were thought to be regulated by the corresponding lncRNAs. (**d**) Enrichment analysis of the mRNAs presented in (**c**). Figure (**a–d**) were drawn by R package (R-4.0.0 Version, https://www.R-project.org)^23^.

To reveal the mechanism of hyperoxia-induced vascular endothelial injury, human LMECs were cultured for 12 and 24 h under 80% hyperoxia (Fig. S1a). The results showed that compared with LMECs in a normal culture environment, the apoptosis of LMECs treated with hyperoxia was significantly enhanced, especially after 24 h (Fig. S1a). Therefore, the treatment with hyperoxia for 24 h was used in the following experiments. The next-generation sequencing revealed that hyperoxia dramatically changed the gene expression profile of LMECs including mRNA, miRNA and lncRNA. The expressions of 607 mRNAs were significantly changed after hyperoxia treatment, of which 152 were up-regulated and 455 were down-regulated (Fig. S1b). The expressions of 80 lncRNAs and 34 miRNAs was also remarkably changed in hyperoxia-treated LMECs (Fig. S1c,d). To reveal the functional implications of the significantly differentially expressed miRNAs, their potential target genes were first predicted with multiple tools. For reducing the false positive rates, the intersection of the prediction results of these softwares was considered as the potential target genes for these miRNAs. Then, the intersection of these potential target genes and the significantly different mRNAs was selected (Fig. S1e). In the second intersection, mRNAs that are significantly negatively correlated with miRNA expression (*p* < 0.05, R < − 0.5) were selected as target genes of these 34 significantly different miRNAs (Fig. 1a). Functional enrichment analysis of these target genes showed that their functions were involved in the intrinsic apoptotic pathway, condensed chromosome, cell cycle and cellular response to oxygen-containing compounds as well as p53, FoxO, PI3K-AKT pathways (Fig. 1b). In addition, many mRNAs regulated by hyperoxia were significantly correlated (*p* < 0.05, |R|> 0.5) or co-expressed with some significantly differentially expressed lncRNAs, which reflected the functional significance of these lncRNAs (Fig. 1c). The functional enrichment analysis showed that these mRNAs were closely related to execution phase of apoptosis, cell cycle, necroptosis, and p53 pathway (Fig. 1d). The above results demonstrated that hyperoxia dramatically changed the gene expression profile of LMECs, and made many functions and signal pathways abnormal, which might be highly associated with hyperoxia-induced endothelial cell apoptosis.

### Hyperoxia significantly regulated the expression of DLEU2, miR-181c-5p and NCAPG and closely correlated with hyperoxia-induced apoptosis in LMEC

**Figure 2:**
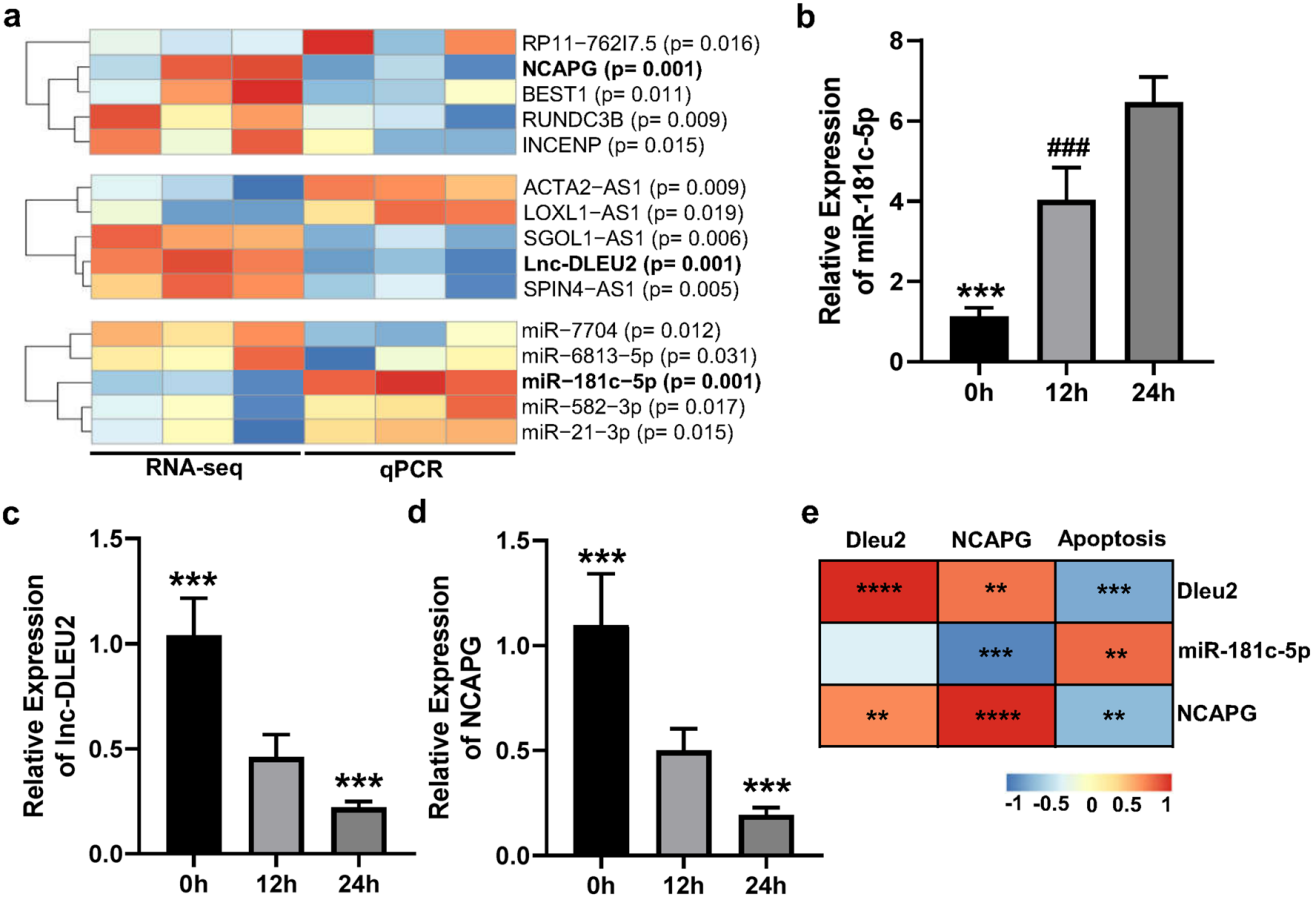
Transcripts most significantly modulated by hyperoxia in LMECs were closely associated with apoptosis. (**a**) Heatmap of the expression levels of the most significant top 5 mRNAs, lncRNAs and miRNAs regulated by hyperoxia in LMECs. (**b**–**d**) Changes in the expression of miR-181c-5p (**b**), lnc-DLEU2 (**c**) and NCAPG (**d**) in LMECs treated with hyperoxia for different times. Data are presented as mean ± SEM. One-way ANOVA analysis with Tukey's post hoc test was used for statistical analysis in (**b**–**d**). ***: corrected *p* < 0.001 versus other groups; ###: corrected *p* < 0.001 versus 24 h groups. (**e**) Heatmap of Spearman’s correlation coefficients between apoptosis rate and the expression levels of miR-181c-5p, lnc-DLEU2 and NCAPG. **: *p* < 0.01; ***: *p* < 0.001; ****: *p* < 0.0001. Figure (**a**,**e**) were drawn by R Software (R-4.0.0 Version, https://www.R-project.org)^23^.

Among these significantly differentially expressed mRNAs, lncRNAs and miRNAs found in the present study, the tendency of the top 5 transcripts with the most significant changes was verified by qPCR, which is consistent with their sequencing results (Fig. 2a). In these transcripts, the expression levels of lincRNA-DLEU2, miR-181c-5p and NCAPG were all significantly changed before and after hyperoxia treatment (Fig. 2b–d) and were also significantly correlated with the hyperoxia-induced apoptosis of LMECs (Fig. 2e). Additionally, there was a significant positive correlation between the expression levels of lincRNA-DLEU2 and NCAPG. In contrast, the expression level of NCAPG was significantly negatively correlated with that of miR-181c-5p (Fig. 2e). These results suggested that there may be a regulatory network among lincRNA-DLEU2, miR-181c-5p and NCAPG, which acted on NCAPG and regulated LMECs apoptosis.

### miR-181c-5p directly targeted NCAPG and inhibited its expression in LMECs

**Figure 3:**
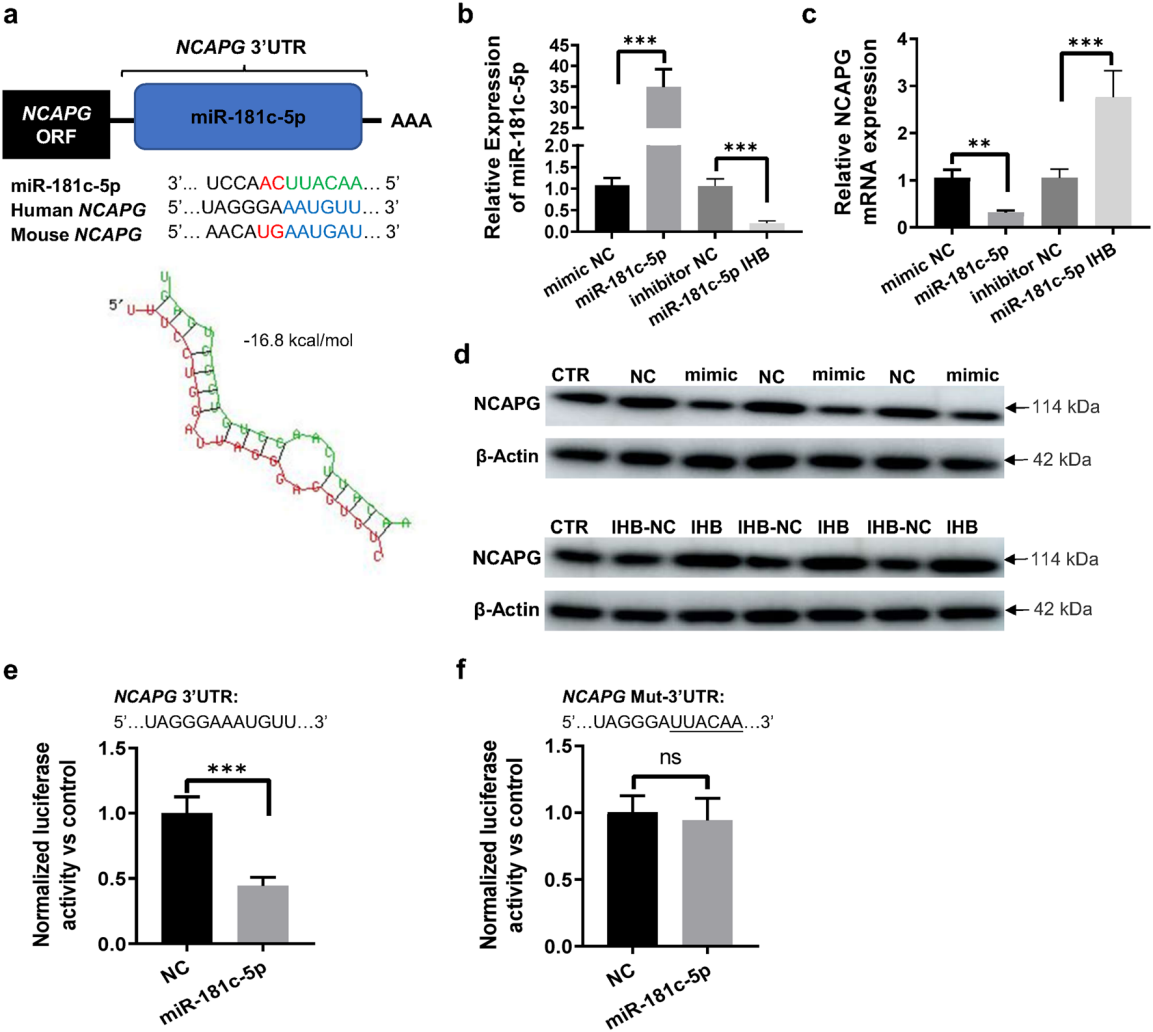
NCAPG was the target gene of miR-181c-5p. (**a**) Upper panel, Graphical representation of the conserved sequences (blue regions) complementary to the seed regions of miR-181c-5p (green regions) in the 3′UTR of NCAPG mRNA of different species. Lower panel, miR-181c-5p could putatively form a strong secondary structure with the target sequence of 3′UTR of NCAPG. Secondary structure was predicted using online tool RNAhybrid^24^. (**b**) The expression level of miR-181c-5p in different groups of LMECs. LMECs were transfected with miR-181c-5p mimic (miR-181c-5p), inhibitor (miR-181c-5p IHB) and the corresponding negative control sequences, respectively. (**c**,**d**) Changes in NCAPG expression at mRNA (**c**) and protein (**d**) levels in different groups of LMECs. LMECs were transfected with miR-181c-5p mimic (mimic), inhibitor (IHB) and the corresponding negative control sequences (NC, IHB-NC). CTR: LMECs without any treatment. (**e**,**f**) Luciferase activity of the luciferase reporter vectors containing either wild-type (**e**) or mutated (**f**) 3′UTR of human NCAPG after miR-181c-5p mimics treatment. The underlined letters are mutated bases in the binding sequence of miR-181c-5p in 3′UTR of NCAPG mRNA. Control: LMECs transfected with empty luciferase reporter vector. NC: LMECs co-transfected with luciferase reporter vector and negative control sequences of miR-181c-5p mimic. miR-181c-5p: LMECs co-transfected with luciferase reporter vector and miR-181c-5p mimic. Data are presented as mean ± SEM. One-way ANOVA analysis with Tukey's post hoc test was used for statistical analysis in (**b,c**). Two-tailed student’s t test was used for statistical analysis in (**e**,**f**). ***: *p* < 0.001; ns: not significant.

Bioinformatics analysis was conducted by using RNAhybrid and Targetscan, which revealed that the binding site of miR-181c-5p seed sequence was present in the 3′UTR of NCAPG. Moreover, this binding site was highly conserved in mammals and had a low binding energy (− 16.8 kcal/mol), suggesting that NCAPG should be the target gene of miR-181c-5p (Fig. 3a). Transfection with miR-181c-5p mimic significantly increased the expression level of miR-181c-5p, while transfection with miR-181c-5p inhibitor down-regulated its expression in LMECs (Fig. 3b). MiR-181c-5p mimic transfection in LMECs significantly downregulated NCAPG, while miR-181c-5p inhibitor transfection markedly upregulated NCAPG at mRNA and protein levels (Fig. 3c,d). The luciferase assay data showed that miR-181-5p mimic transfection significantly decreased the luciferase activity of dual-luciferase reporter vector containing the wild miR-181c-5p binding sites in the 3′UTR of NCAPG mRNA in LMECs. However, the luciferase activity had not changed significantly in LMECs co-transfected with miR-181c-5p mimics and dual-luciferase reporter vector containing the mutant binding sites for miR-181c-5p (Fig. 3e,f).

### miR-181c-5p promoted hyperoxia-induced apoptosis by inhibiting NCAPG expression in LMECs

**Figure 4:**
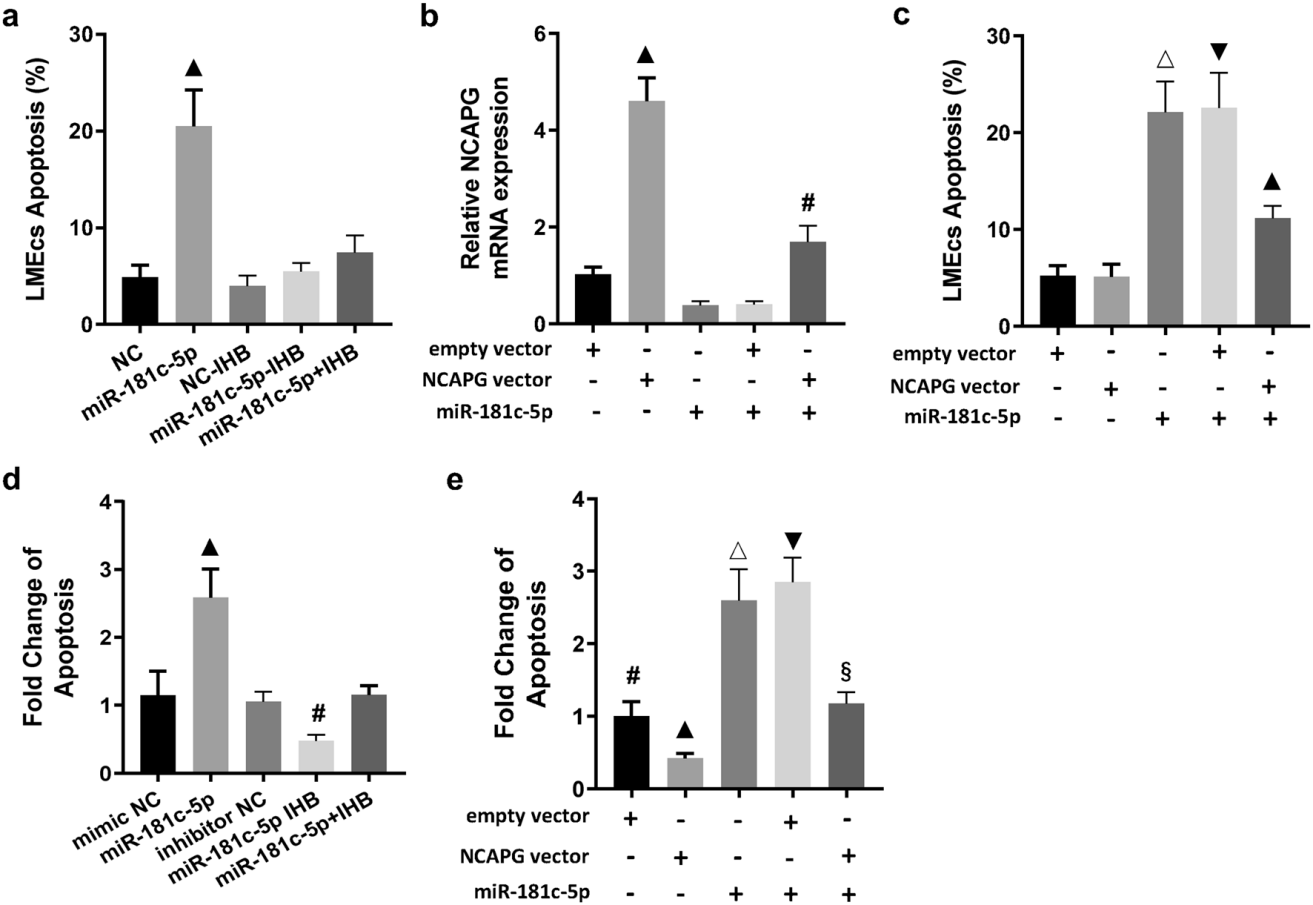
MiR-181c-5p promoted hyperoxia-induced apoptosis of LMECs by inhibiting the expression of its target gene NCAPG. (**a**) Comparison of apoptosis of LMECs treated with miR-181c-5p mimic (miR-181c-5p) or inhibitor (IHB) and the corresponding negative control sequences (NC, NC-IHB). **▲**: *p* < 0.001 versus all other groups. (**b**) Comparison of expression levels of NCAPG mRNA in LMECs with different treatments. **▲**: *p* < 0.001 versus all other groups; **#**: *p* < 0.01 versus all other groups. (**c**) Comparison of apoptosis of LMECs with different treatments. △: *p* < 0.001 versus all other groups except for miR-181c-5p + empty vector group; ▼: *p* < 0.001 versus all other groups except for miR-181c-5p group; **▲**: *p* < 0.001 versus all other groups. (**d**) Relative changes of apoptosis of hyperoxia-treated LMECs transfected with miR-181c-5p mimic (miR-181c-5p) or inhibitor (IHB) and the corresponding negative control sequences (NC, IHB-NC). **▲**: *p* < 0.001 versus all other groups; #: *p* < 0.01 versus all other groups. (**e**) Relative changes of apoptosis of hyperoxia-treated LMECs transfected with miR-181c-5p or different vectors. #: *p* < 0.01 versus all other groups; **▲**: *p* < 0.001 versus all other groups; △: *p* < 0.001 versus all other groups except for miR-181c-5p + empty vector group; ▼: *p* < 0.001 versus all other groups except for miR-181c-5p group; **§**: *p* < 0.001 versus all other groups except for empty vector group. Data are presented as mean ± SEM. One-way ANOVA analysis with Tukey's post hoc test was used for statistical analysis.

In order to clarify the role and mechanism of miR-181c-5p in endothelial injury induced by hyperoxia, mimic and inhibitor of miR-181c-5p and its corresponding negative control sequences were transfected into LMECs respectively. The results showed that miR-181c-5p significantly increased the apoptosis of LMECs compared with the control sequence, while miR-181c-5p inhibitor abrogated the pro-apoptotic effect of miR-181c-5p mimic. However, miR-181c-5p inhibitor transfection alone did not significantly affect the apoptosis of LMECs (Fig. 4a). For clarifying whether miR-181c-5p could promote the apoptosis of LMECs through targeting NCAPG, the expression vector of NCAPG and its empty vector were co-transfected with miR-181c-5p mimic to LMECs respectively. The results showed that the NCAPG expression vector rescued the down-regulation of NCAPG expression caused by miR-181c-5p and significantly decreased the miR-181c-5p-induced apoptosis of LMECs, while the empty vector had no such effect (Fig. 4b,c). MiR-181c-5p significantly enhanced the apoptosis of LMECs induced by hyperoxia, while miR-181c-5p inhibitor abrogated the pro-apoptotic effect of miR-181c-5p. Moreover, miR-181c-5p inhibitor transfection alone also significantly attenuated hyperoxia-induced apoptosis of LMECs (Fig. 4d). Additionally, NCAPG expression vector also abrogated the promotive effect of miR-181c-5p on hyperoxia-induced apoptosis of LMECs (Fig. 4e). The above results fully indicated that miR-181c-5p promoted hyperoxia-induced apoptosis of LMECs through targeted inhibition of NCAPG expression.

### lncRNA-DLEU2 directly bind to miR-181c-5p and negatively regulated its effects

**Figure 5:**
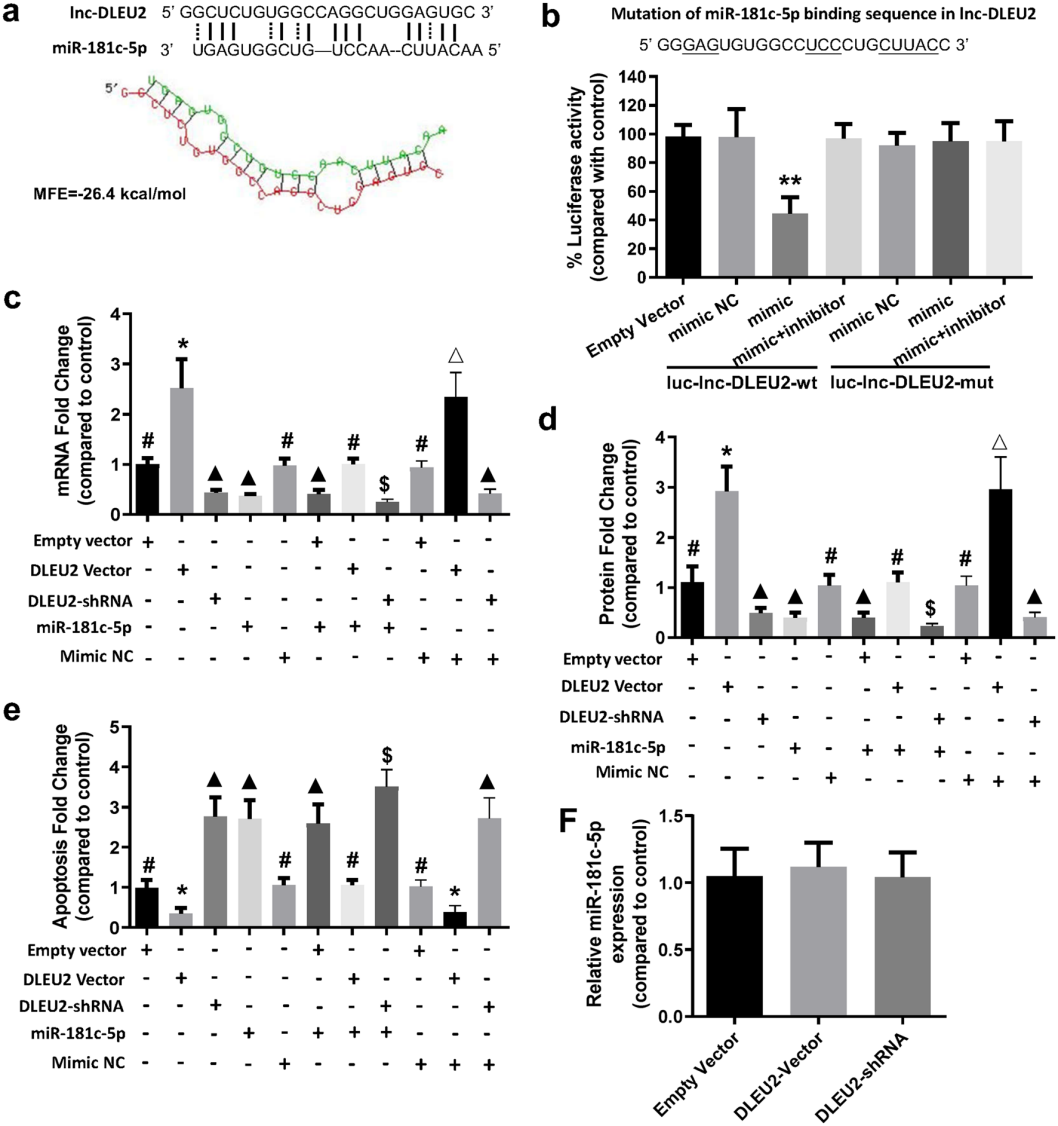
LncRNA-DLEU2 directly binds to miR-181c-5p and inhibited its effects on NCAPG expression and apoptosis of LMECs. (**a**) Upper panel, the predicted core binding sequence of miR-181c-5p in lnc-DLEU2 sequence. The solid line represented the standard base pairing and strong interaction; dashed line represented the non-standard base pairing and relatively weak interaction. Lower panel, the predicted secondary structure of the binding site of miR-181c-5p and lnc-DLEU2. Secondary structure was predicted using online tool RNAhybrid^24^. (**b**) Upper panel, the mutation of miR-181c-5p binding sequence in lnc-DLEU2. The underlined letters indicated the mutation bases. Lower panel, LMECs was co-transfected by no-insert empty vector, luc-lnc-DLEU2-wt vector or luc-lnc-DLEU2-mut vector along with miR-181c-5p (mimic), or mimic and inhibitor of miR-181c-5p (mimic + inhibitor), or negative control sequences (mimic NC). **: *p* < 0.01 compared with all other groups. All experiments were repeated five times independently. (**c**,**d**) The relative expression levels of NCAPG at mRNA (**c**) and protein (**d**) levels in LMECs with different treatments. #: *p* < 0.01 versus all other groups except for group with the same label; *: *p* < 0.001 versus all other groups except for DLEU2 Vector + Mimic NC group; **△**: *p* < 0.001 versus all other groups except for DLEU2 Vector group; **▲**: *p* < 0.01 versus all other groups except for group with the same label; $: *p* < 0.01 versus all other groups. (**e**) Relative changes of apoptosis of LMECs with different treatments. The untreated LMECs were used as control. #: *p* < 0.01 versus all other groups except for group with the same label; *: *p* < 0.01 versus all other groups except for group with the same label; **▲**: *p* < 0.01 versus all other groups except for group with the same label; **$**: *p* < 0.05 versus all other groups. (**f**) The relative expression levels of miR-181c-5p in LMECs with different treatments. The non-transfected LMECs were used as control in (**c**–**f**). Data are presented as mean ± SEM. One-way ANOVA analysis with Tukey's post hoc test was used for statistical analysis.

Lnc-DLEU2 is located on chromosome 13q14. Although lnc-DLEU2 has been reported to play important roles in various tumors, its function in oxygen toxicity and endothelial cells has not been described thus far. According to our aforementioned results, there might be a regulatory network among lincRNA-DLEU2, miR-181c-5p and NCAPG. Considering that lncRNA can act as a miRNA sponge, we speculated that lnc-DLEU2 was likely to bind miR-181c-5. The predicted results using RNAhybrid also showed that lnc-DLEU2 could bind to miR-181c-5p. The MFE between miR-181c-5p and the miR-181c-5p binding site in lnc-DLEU2 was − 26.4 kcal/mol (Fig. 5a). To further confirm the binding ability between the them, the luciferase reporter assay was conducted. The results showed that only miR-181c-5p mimic significantly reduced the relative luciferase activity in LMECs transfected with lnc-DLEU2-wt. The luciferase activity in other groups did not significantly change (Fig. 5b). This finding indicated that lnc-DLEU2 could bind to miR-181c-5p and might act as a sponge to suppress the inhibitory effects of miR-181c-5p on its target genes. To validate this conclusion, lnc-DLEU2 expression vector and shRNA vector and their corresponding empty vectors were constructed and transfected into LMECs alone or with miR-181c-5p. The results showed that lnc-DLEU2 expression vector significantly blocked the inhibitory effect of miR-181c-5p on the expression of its target gene NCAPG at the mRNA and protein levels, as well as the pro-apoptotic effect of miR-181c-5p on LMECs, while the shRNA vector of lnc-DLEU2 had the opposite effect and empty vector had no significant effect (Fig. 5c–e). In addition, the transfection of expression vector or shRNA vector of lnc-DLEU2 all had no significant effect on the expression level of miR-181c-5p in LMECs (Fig. 5f). These results indicate that lnc-DLEU2 suppressed the inhibitory effects of miR-181c-5p on its target NCAPG as a sponge of miR-181c-5p.

### Lnc-DLEU2 inhibited the LMEC apoptosis induced by hyperoxia through a ceRNA network consisted of DLEU2, miR-181c-5p and NCAPG

**Figure 6:**
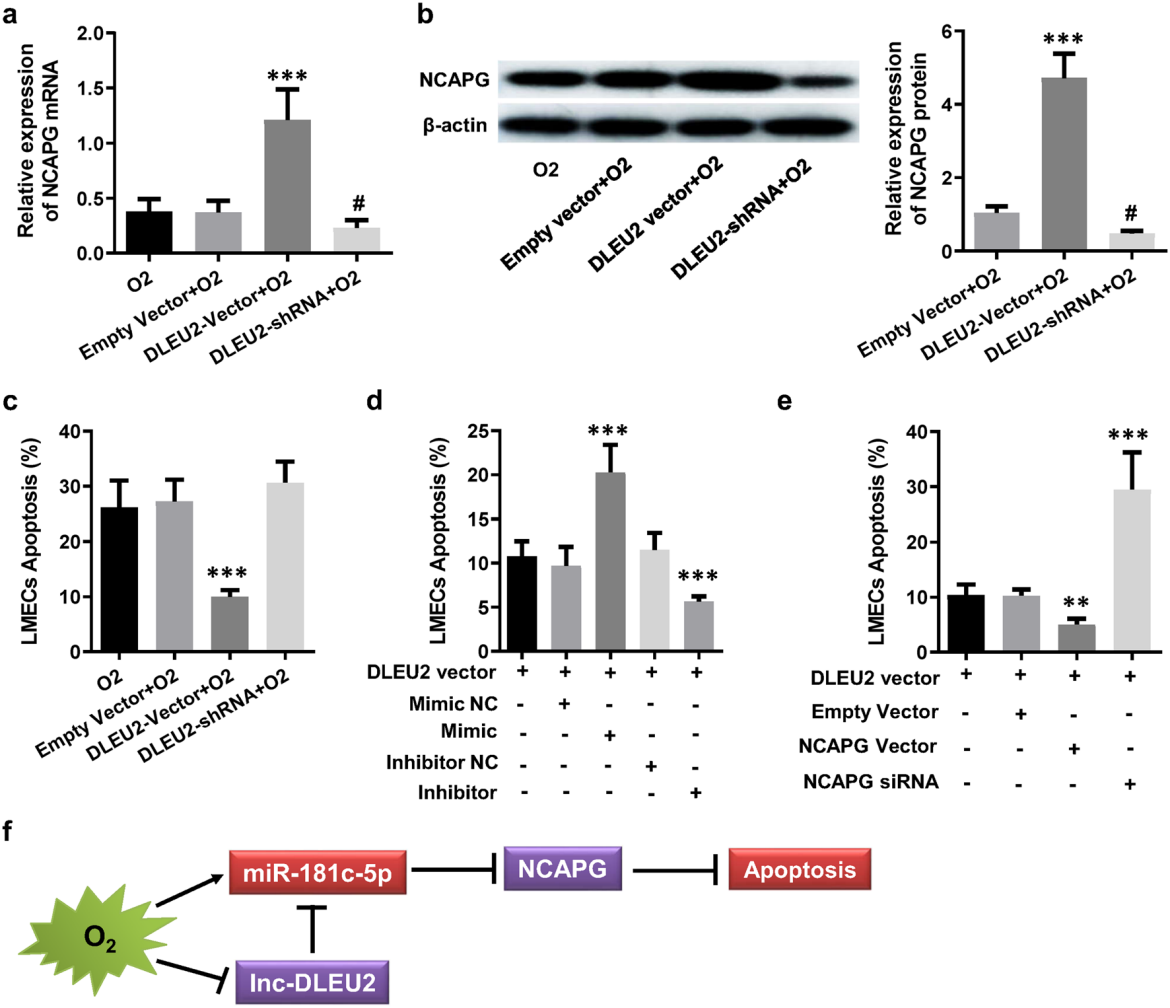
Lnc-DLEU2 inhibited hyperoxia-induced apoptosis of LMECs through a ceRNA network. (**a**,**b**) The relative expression levels of NCAPG at mRNA (**a**) and protein (**b**) levels in hyperoxia-treated LMECs with different treatments compared to the untreated (**a**) or hyperoxia-treated (**b**) LMECs. ***: *p* < 0.001 versus all other groups; #: *p* < 0.05 versus all other groups. (**c**) Relative changes of apoptosis of hyperoxia-treated LMECs with different treatments compared to the untreated LMECs. ***: *p* < 0.001 versus all other groups. (**d**) Relative changes of apoptosis of hyperoxia-treated LMECs transfected with lnc-DLEU2 vector, miR-181c-5p mimic and/or inhibitor as well as the corresponding negative control sequences. The untreated LMECs were used as control. ***: *p* < 0.001 versus all other groups. (**e**) Relative changes of apoptosis of hyperoxia-treated LMECs transfected with lnc-DLEU2 vector, and/or NCAPG vectors or siRNA. The untreated LMECs were used as control. **: *p* < 0.01 versus all other groups. ***: *p* < 0.001 versus all other groups. (**f**) The schematic diagram of the pro-apoptotic effects of miR-181c-5p through a ceRNA network consisted of lnc-DLEU2, miR-181c-5p and NCAPG in LMECs. The red rectangle indicated upregulated, blue rectangle indicted downregulated. Data are presented as mean ± SEM. One-way ANOVA analysis with Tukey's post hoc test was used for statistical analysis.

To further elucidate the relationship among miR-181c-5p, NCAPG and lnc-DLEU2 in endothelial injury caused by hyperoxia, lnc-DLEU2 expression vector and shRNA vector or their corresponding empty vectors were transfected into hyperoxia-treated LMECs. The results showed that lnc-DLEU2 expression vector remarkably attenuated the down-regulation of NCAPG mRNA and protein caused by hyperoxia, while lnc-DLEU2 shRNA vector had the opposite effect on NCAPG expression, and the empty vector had no significant effect on NCAPG expression (Fig. 6a,b). Meanwhile, the lnc-DLEU2expression vector could markedly attenuated hyperoxia-induced apoptosis of LMECs, while the lnc-DLEU2 shRNA vector had the opposite effect, and the empty vector has no significant effect (Fig. 6c). Additionally, miR-181c-5p mimic transfection significantly reduced the inhibitory effect of lnc-DLEU2 expression vector on hyperoxia-induced apoptosis of LMECs, while miR-181c-5p inhibitor enhanced this effect (Fig. 6d). Furthermore, the siRNA of NCAPG dramatically reduced the inhibitory effect of lnc-DLEU2 expression vector on hyperoxia-induced apoptosis of LMECs, but NCAPG expression vector significantly enhanced it (Fig. 6e). The above results illustrated that miR-181c-5p and its target gene NCAPG as well as DLEU2 constituted a ceRNA network, which played critical roles in the apoptosis of LMECs induced by high-concentration oxygen (Fig. 6f).

## Discussion

In the present study, we found that hyperoxia profoundly affected the functions and signaling pathways of LMECs. Hyperoxia significantly downregulated lncRNA-DLEU2 expression, thereby abrogating its inhibitory effect on miR-181c-5p as a sponge and decreasing the expression of target gene of miR-181c-5p (NCAPG), finally promoted apoptosis of LMECs. This ceRNA network consisting of lncRNA-DLEU2, miR-181c-5p and NCAPG revealed by our study will help to understand the molecular mechanisms for the vascular endothelial damage caused by hyperoxia.

In clinical practice, high-concentration oxygen inhalation is the most commonly used method of oxygen therapy. Therefore, the lungs, especially the pulmonary capillaries, are one of the primary targets of damage during prolonged high-concentration oxygen therapy because of oxidative stress induced by excessive O_2_^25^. Several studies have reported the molecular mechanism of hyperoxemia damage to vascular endothelium^26^. The abnormality of TLR4-STC1-mediated mitochondrial pathway is thought to be an important mechanism of hyperoxia-induced lung endothelium injury^27^. Hyperoxia can also activate p47phox and mediate lung endothelium injury through Spns2/S1P/S1P1&2 signaling axis^28^. TLR4-VEGFR2 pathway plays important roles in protection against hyperoxia-induced injury of lung endothelial cells^29^. Nevertheless, we still have limited knowledge about the pathophysiologic mechanisms in endothelial cell injury induced by hyperoxemia.

Numerous studies have shown lncRNAs play essential roles in lung injury induced by hyperoxia through the ceRNA mechanism. For example, lncRNA gadd7 could bind to miR-125a and regulate the expression of its target gene, MFN1, in small cuboidal cells of alveoli, thereby plays critical roles in the protective effect of agmatine against hyperoxia-induced acute lung injury^30^. Long noncoding RNA FOXD3-AS1 could exaggerate hyperoxia-induced lung epithelial cell death as a sponge or competing endogenous noncoding RNA for miR-150^31^. Nevertheless, there are still contradictions regarding the detailed roles of certain lncRNAs. Some studies showed that lncRNA MALAT1 promoted BPD induced by hyperoxia through interacting with CREB^32^. However, other studies showed that lncRNA MALAT1 protected preterm infants with BPD by inhibiting cell apoptosis^33^. Our study revealed for the first time that lncRNA-DLEU2 played a protective role in hyperoxia-induced apoptosis of LMECs by acting as a sponge or competing endogenous noncoding RNA for miR-181c-5p. All the results suggested that lncRNA is the important regulator of hyperoxia-induced endothelial injury and deserves further investigation.

Accumulating evidences have demonstrated that miRNAs are strongly associated with hyperoxia-induced endothelial dysfunction. For example, the expressions of many miRNAs were significantly changed in alveolar of the neonatal rat model of hyperoxia-induced BPD^34^. MiRNA-451 expression was significantly upregulated in mouse lung endothelial cells exposed to hyperoxia, which was involved in the development of BDP^35^. MiR-34a can induce mitochondrial-mediated apoptosis by inhibiting the expression of its target gene SIRT1, thereby promoting hyperoxia-induced endothelial dysfunction^36^. The differential expression of miR-30a in human pulmonary microvascular endothelial cells contributed to protection from hyperoxic lung injury in female neonatal mice through decreased Snai1 expression^37^. Our study found that miR-181c-5p could promote hypoxia-induced apoptosis in LMECs by inhibiting the expression of its target gene, NCAPG. These studies, including ours, suggest that miRNAs play important roles in hypoxia-induced apoptosis of vascular endothelium, and might be valuable targets for preventing hyperoxia injury.

As an important lncRNA, lnc-DLEU2 was reported to be associated with the development and prognosis of various tumors^38,39^. However, there are also contradictory findings. Some studies showed that histone deacetylases inhibitor trichostatin A treated non-small cell lung cancer through upregulation of Dleu2/miR-15a/16-1^40^. But other studies suggested that lncRNA DLEU2 exhibited tumorigenic effects through downregulating the inhibitory effect of miR-30c-5p on SOX9 expression in non-small cell lung cancer^41^. To the best of our knowledge, no studies on the function of lnc-DLEU2 in endothelial cells have been reported so far. Our study showed that lnc-DLEU2 could inhibit apoptosis of LMECs and exert an oncogenic-like effect through the mechanism of ceRNA, which is basically consistent with previous studies reported.

Many studies have shown that miR-181c-5p has multiple functions, such as regulating cell apoptosis, inflammation, immunity, and mitochondrial function. MiR-181c-5p could down-regulate PTPN4 to promote the inflammatory response during ischemia/reperfusion injury, and also could regulates ischemia/reperfusion injury-induced neuronal cell death by targeting c-Fos^42,43^. Nevertheless, miR-181c-5p remains poorly studied in vascular endothelial cells. Recent studies have found that MiR-181c-3p and miR-181c-5p could significantly enhance high glucose-induced oxidative stress injury and apoptosis of human umbilical vein endothelial cells (HUVECs) through their target LIF^44^. Our study found that hyperoxia significantly up-regulated miR-181c-5p expression in vascular endothelial cells and promoted apoptosis, which is an important mechanism for hyperoxia to cause endothelial damage. This negative regulatory effect of miR-181c-5p on endothelial cell function is consistent with previous studies^44^.

NCAPG is a subunit of condensin I, a large protein complex involved in chromosome condensation. NCAPG overexpression may play important roles in carcinogenesis, drug resistance and progression of tumors via regulating tumor-related pathways^45,46^. Many studies have demonstrated that NCAPG should be considered as an essential oncogene in hepatocellular carcinoma^47^. For instance, miR-181c expression was significantly decreased in hepatocellular carcinoma and exhibited tumor-suppression via targeting NCAPG^48^. Our study indicated that NCAPG also exerted an oncogene-like function in vascular endothelial cells as it did in many tumors. This finding revealed for the first time the function of NCAPG in vascular endothelial cells and contributed to a deeper understanding of the molecular mechanisms of endothelial cell injury.

In conclusion, this study systematically revealed the effects of hyperoxia on the transcriptome of pulmonary capillary endothelial cells and reported for the first time the roles of a ceRNA network composed of miR-181c-5p, lncRNA DLEU2 and NCAPG in hyperoxia-induced endothelial injury. Our findings provide valuable clues for a deeper understanding of the mechanism of hyperoxia-induced damage. However, it should be pointed out that this study still has some shortcomings. For example, this research was conducted at the cellular level in vitro. Further in vivo studies are needed to determine whether the molecular mechanisms revealed in the present study play the same roles at the animal level. Additionally, the molecular mechanism by which NCAPG inhibits hyperoxia-induced apoptosis of endothelial cells also needs to be further investigated. Nevertheless, we believe that this study will contribute to the full understanding of the harmful effects of hyperoxia and to effectively mitigate its deleterious effects.

## Supplementary Information

Supplementary Information.
