# Supplementary material for: miR-181c-5p mediates apoptosis of vascular endothelial cells induced by hyperoxemia via ceRNA crosstalk
Source: Sci Rep. 2021 Aug 16;11:16582. doi: 10.1038/s41598-021-95712-1 (PMC8368219; doi:10.1038/s41598-021-95712-1)
Supplement: Supplementary file 1 — Supplementary Information. [file 41598_2021_95712_MOESM1_ESM.docx]

**miR-181c-5p mediates apoptosis of vascular endothelial cells induced by hyperoxemia via ceRNA crosstalk**

Jizhi Wu; Guangqi Zhang; Hui Xiong; Yuguang Zhang; Gang Ding; Junfeng Ge^*^

**Supplementary Figure Legends**


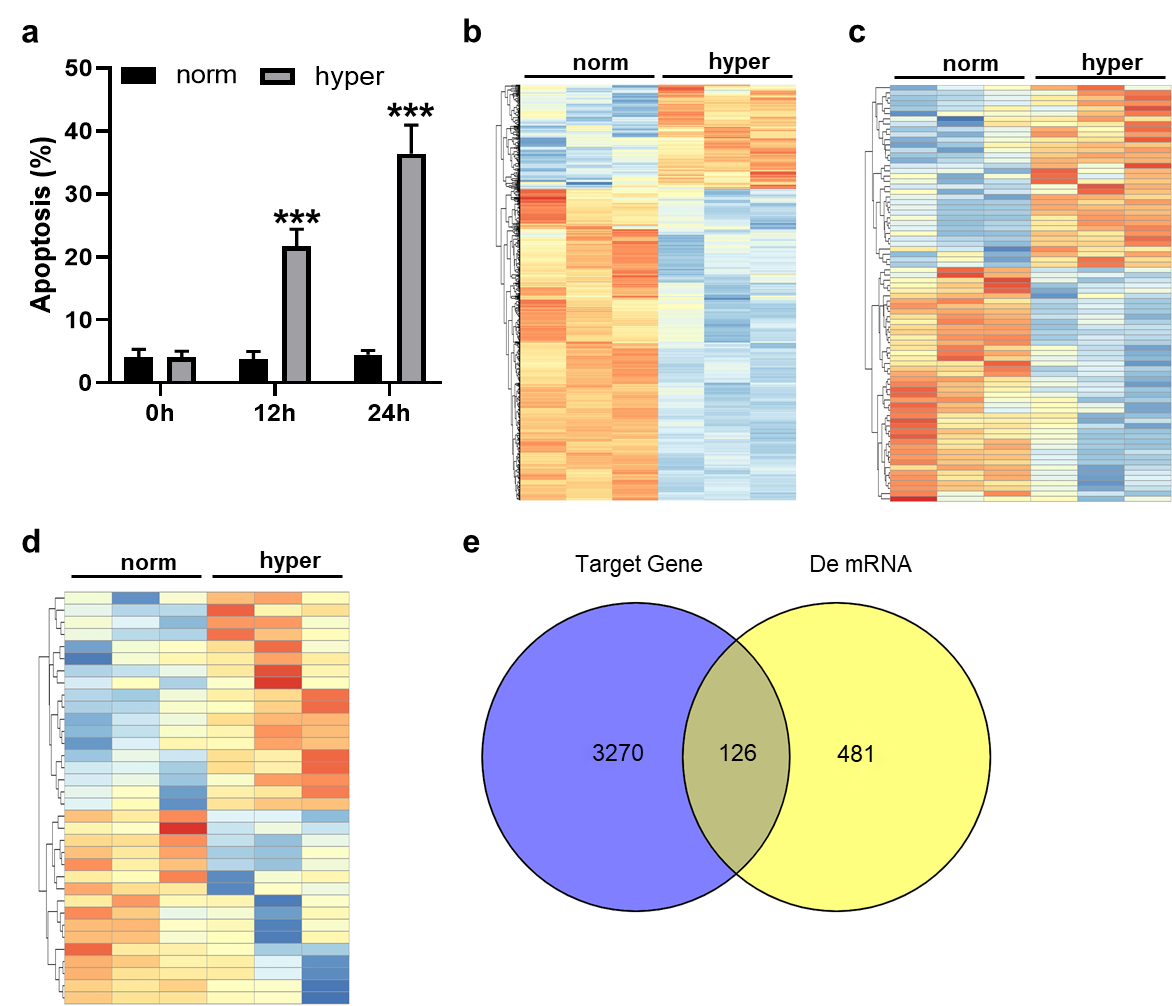


**Figure S1. (a)** The apoptosis rates of LMECs treated with normoxie (norm) or hyperoxie (hyper) for 0 h, 12 h and 24 h. *******: *p* < 0.001 vs. all other groups. **(b-d)** Heatmap of the standardized expression levels of the significantly differentially expressed mRNAs (b), lncRNAs (c) and miRNAs (d). **(e)** Venn diagram of the intersection of the potential target genes of the 34 significantly differentially expressed miRNAs and the significantly differentially expressed mRNAs (De mRNA). Venn diagram was drawn using online tool venny 2.1 (https://bioinfogp.cnb.csic.es/tools/venny/index.html).


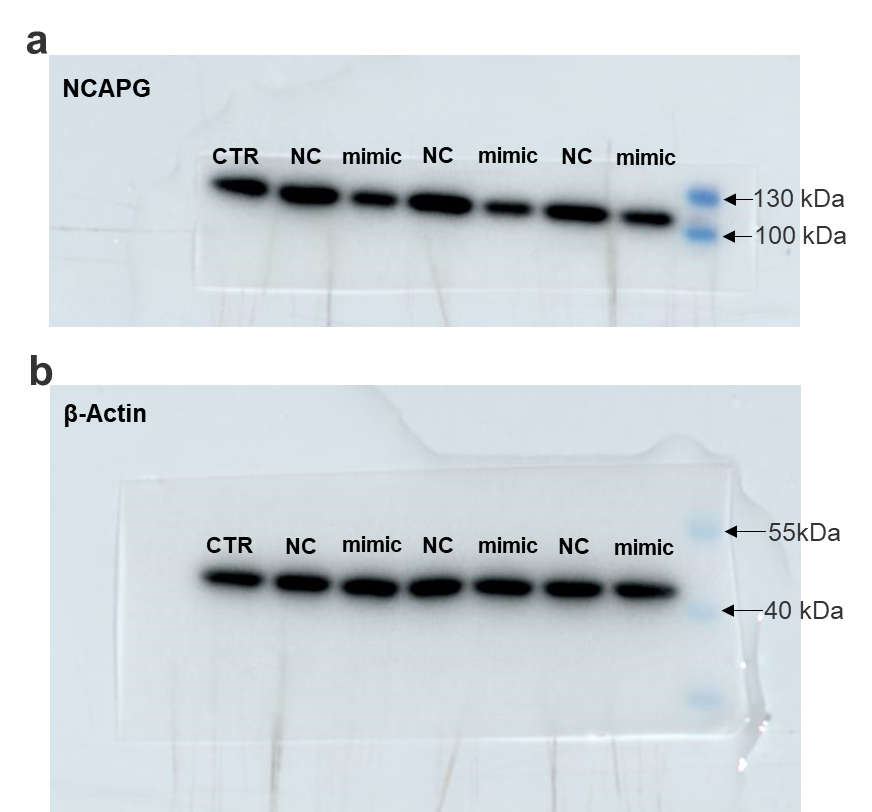


**Figure S2.** The full-size images of Western Blot for NCAPG protein (a) and β-actin (b) in Figure 3D (upper panel).


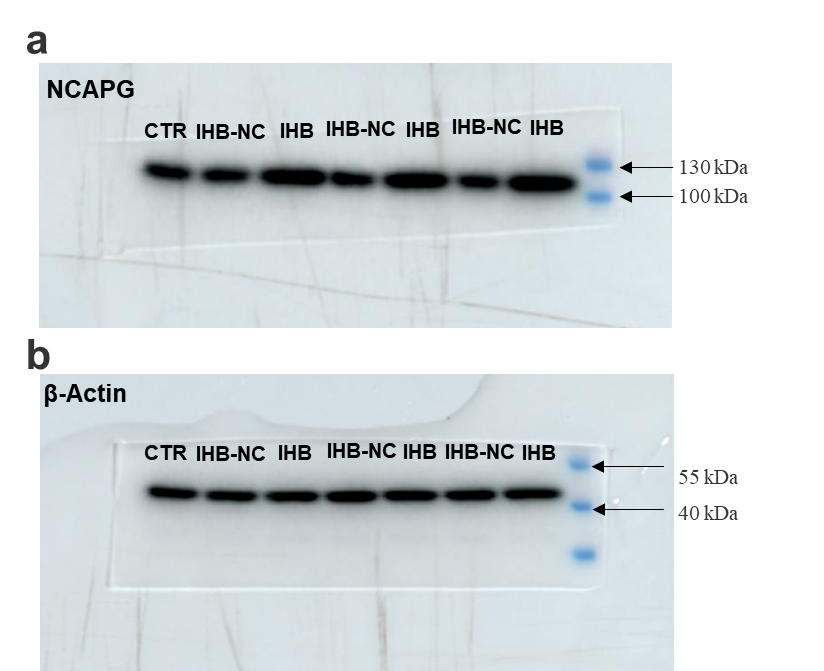


**Figure S3.** The full-size images of Western Blot for NCAPG protein (a) and β-actin (b) in Figure 3D (lower panel).


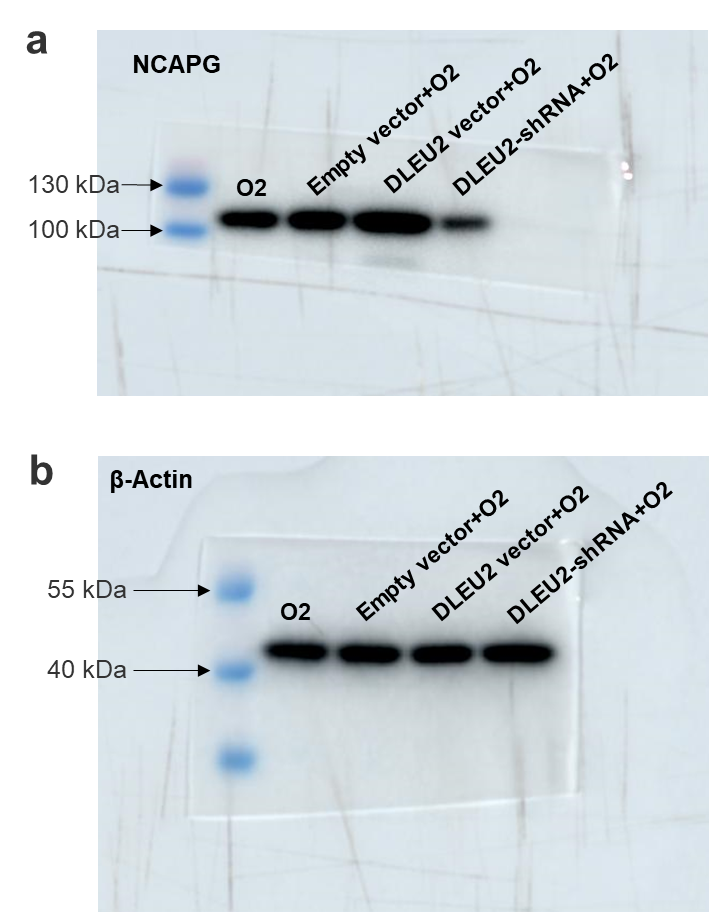


**Figure S4.** The full-size images of Western Blot for NCAPG protein (a) and β-actin (b) in Figure 6B.
